# Medical Case Reporting for Pancreatic Cancer

**DOI:** 10.1089/crpc.2015.29007.cjy

**Published:** 2016-01-01

**Authors:** Charles J. Yeo, Jordan L. Schilling

**Affiliations:** ^1^ Department of Surgery, Thomas Jefferson University, Philadelphia, Pennsylvania.; ^2^ Mary Ann Liebert, Inc., New Rochelle, New York.

2016 will be the start of our second volume of *Case Reports in Pancreatic Cancer* and we could not be more excited for the new year. Our launch in November 2015 was warmly received, and we collaborated with Pancreatic Cancer Action Network's #WageHope campaign on Twitter. Additionally, we celebrated our official launch at the 10th Annual Pancreatic Cancer and Related Diseases Symposium along with Thomas Jefferson University, the Sidney Kimmel Cancer Center, and the Pancreatic Cancer Action Network.

Even with a new year ahead of us, we still have many challenges ahead of us. Pancreatic cancer poses an enormous challenge to clinicians and cancer scientists. Despite efforts in the past 60 years, conventional treatments such as surgery and chemotherapy are not consistently successful. Pancreatic cancer rates have risen steadily for several years, and the disease results in more than 300,000 deaths globally each year. It is the fourth most common cause of cancer deaths in the United States and has a 5-year survival rate of less than 10% when considering all cases. Case reports offer an important opportunity to transfer medical knowledge and stimulate new ideas about clinical care and research direction. More medical reporting is needed to increase the medical knowledge of pancreatic cancer with the aim of leading to significant therapeutic and prognostic progress.

It is out of this need for medical reporting that we launched *Case Reports in Pancreatic Cancer* as a premier online open access journal for clinicians and cancer scientists to report their medical experiences. We hope that *Case Reports in Pancreatic Cancer* will promote interest in pancreatic cancer and encourage medical reporting among researchers, physicians, surgeons, medical oncologists, radiation oncologists, gastroenterologists, and pathologists.

Finally, we have recently added several new Associate Editors, some being international experts, to our expanding editorial board. We are looking forward to continued development of the Journal. Please reach out to us for more information. We look forward to your contribution.

